# Mechanism of Hexane Displaced by Supercritical Carbon Dioxide: Insights from Molecular Simulations

**DOI:** 10.3390/molecules27238340

**Published:** 2022-11-29

**Authors:** Jiasheng Song, Zhuangying Zhu, Lang Liu

**Affiliations:** Key Laboratory of Low-Grade Energy Utilization Technologies and Systems, School of Energy and Power Engineering, Chongqing University, Ministry of Education, Chongqing 400044, China

**Keywords:** molecular simulations, hexane, CO_2_, displacing efficiency, injection pressure

## Abstract

Supercritical carbon dioxide (sCO_2_) has great potential for displacing shale oil as a result of its high solubility and low surface tension and viscosity, but the underlying mechanisms have remained unclear up to now. By conducting equilibrium molecular dynamics (EMD) simulations, we found that the displacing process could be divided into three steps: the CO_2_ molecules were firstly injected in the central region of shale slit, then tended to adsorb on the SiO_2_-OH wall surface and mix with hexane, resulting in loose hexane layer on the shale surface, and finally displaced hexane from the wall due to strong interactions between CO_2_ and wall. In that process, the displacing velocity and efficiency of hexane exhibit parabolic and increased trends with pressure, respectively. To gain deep insights into this phenomenon, we further performed non-equilibrium molecular dynamics (NEMD) simulations and found that both the Onsager coefficients of CO_2_ and hexane were correlated to increase with pressure, until the diffusion rate of hexane being suppressed by the highly dense distribution of CO_2_ molecules at 12 MPa. The rapid transportation of CO_2_ molecules in the binary components (CO_2_ and hexane) actually promoted the hexane diffusion, which facilitated hexane flowing out of the nanochannel and subsequently enhanced oil recovery efficiency. The displacing process could occur effectively at pressures higher than 7.5 MPa, after which the interaction energies of the CO_2_-wall were stronger than that of the hexane-wall. Taking displacing velocity and efficiency and hexane diffusion rate into consideration, the optimal injection pressure was found at 10.5 MPa in this work. This study provides detailed insights into CO_2_ displacing shale oil and is in favor of deepening the understanding of shale oil exploitation and utilization.

## 1. Introduction

In the past decade, the global consumption of fossil energy has tremendously increased, which increases the urgency of the development of new hydrocarbon resources [1,2,3]. Among the unconventional sources, shale oil has aroused extensive interest attributed to its abundant reserves, continuous distribution, and easy exploitation in comparison with the existing primary fossil energy [4,5,6]. Shale oil is widely presented in nano-porous shale slit through physicochemical changes such as adsorption and deposition [7,8,9,10]. Some technologies such as horizontal drilling, hydraulic fracturing, and supercritical carbon dioxide (sCO_2_) displacing methods have been developed to exploit shale oil [1,11,12]. The method of sCO_2_ displacing shale oil has some unique advantages, such as high solubility, swelling shale oil, low surface tension and viscosity, and enhanced crude oil recovery, compared with other technologies [13,14]. In consideration of the high efficiency involved in extracting shale oil and weakening greenhouse effects, the sCO_2_ displacing shale oil technology has developed rapidly in recent years [12,15,16,17]. However, the underlying mechanisms of the displacing process involved have remained unclear up to now.

Some studies have compared the performance of shale oil displacing by various components, and summarized common rules in the displacing process. For instance, Zhang et al. [18] investigated the adsorption and diffusion of shale oil in nanopores using molecular dynamics (MD) simulations. They found that the oil adsorption capacity followed the sequential order of organic matter > inorganic material > montmorillonite, and the diffusivities of octane in the shale nanopores were on the order of 10^−9^ m^2^/s. Zhong et al. [19] adopted four components with different polarities including decane, methyl benzene, pyridine and acetic acid in crude oil to reveal the adsorption behaviors of different sorbates on a silica surface. They found that the adsorption capability of the adsorbent was closely associated with interactions between the adsorbate and the silica surface. The polar oil components were preferentially absorbed on the mineral surface, which acted as an anchor to promote the adsorption of nonpolar counterparts.

Attributed to the essential and fundamental phenomenon, the flow and diffusion behaviors for shale gases in nano-porous shale have aroused wide attentions. By using simulations, Chen et al. [20] investigated the flow and transport of shale gas in a nano-porous shale structure. They found that shale with high tortuosity demonstrated extremely low intrinsic permeability, whereas the diffusion mode was dominated by Knudsen diffusion. In addition, the transport process was also taken into consideration by Wang et al. [21], who revealed the flow characteristics of CH_4_ molecules transport through calcite nanopores, and found that the slower mass transfer arose from strong interactions and the rough inner surface.

Extensive efforts have also been devoted to the displacing process of shale oil for exploring useful methods in enhancing oil recovery. Shuai et al. [22] investigated the sCO_2_ extraction process of organic matter from shale oil by experiments and simulations. They revealed that the displacement interface was more centralized as injection flow and residence increased, which could further enhance displacement rates. Lara et al. [23] studied the effect of improved oil recovery (IOR) agents, including NaCl solutions, CO_2_, N_2_, and CH_4_ on IOR process by MD simulations. They found that CO_2_ outperformed other components since its miscibility increased with pressure. Attributed to low interfacial tension and rapid diffusion in the oil phase, sCO_2_ was especially suitable for IOR applications. Similar conclusions were also drawn by Nguyen et al. [24], who compared the performance of three different injection fluids, including sCO_2_, N_2_, and water in oil recovery, and found that sCO_2_-based recovery approached 90% and outperformed the other two fluids. Seyyedi et al. [25] studied the interactions between carbonated water injection (CWI) and live crude oil through experimentation. The results revealed that CO_2_ was gradually transferred to the oil, which liberated lean gases and improved oil recovery in the CW-oil system. However, the mutual mass transfer between CO_2_ and oil in the CO_2_-oil system led to heavier oil than the original. Lashgari et al. [15] revealed the dominant mechanisms of improved oil recovery in the injection of miscible enriched gas and CO_2_. They found that the diffusion could enhance CO_2_ flux delivery and contribute to oil recovery efficiency.

Apart from the essential role of the diffusion process, pressure and temperature both have an enormous influence on the displacing outcome. Fakher et al. [12] investigated oil recovery and carbon capture from shale reservoirs in the CO_2_ injection process. They found that cyclic CO_2_ injection had strong potential in enhancing oil recovery. The increasing pressure in conjunction with low temperature were both acted to improve oil recovery and CO_2_ storage. Similar results were also obtained by Elwegaa et al. [11], who studied oil recovery from shale oil reservoirs by using cyclic cold CO_2_ and found that the cold CO_2_ injection enhanced both porosities and permeabilities of the core samples up to 3.5% and 8.8%, leading to a higher oil recovery factor in comparison with that at ambient temperature.

As reviewed above, the current studies mainly concentrated on adsorption and diffusion behaviors in nano-porous shale, but placed insufficient emphasis on the shale oil displacing process by sCO_2_. Since the huge advantages of this technology in exploiting shale oil, the underlying mechanisms, especially the detailed displacing process, injection pressure, and mutual interference should be revealed urgently. In this study, the hydroxylated silica and hexane were adopted as shale slit and shale oil, respectively. We revealed the process of sCO_2_ displacing hexane, searching for the optimal pressure by taking the displacing velocity and efficiency, as well as the displacing mechanisms in the presence of sCO_2_ and hexane into consideration. This study provides new insights in sCO_2_ displacing shale oil, and supports the deepening of the understanding of shale oil exploitation and utilization.

## 2. Results and Discussions

### 2.1. CO_2_ Displacing Hexane from SiO_2_-OH Wall

**Figure 1:**
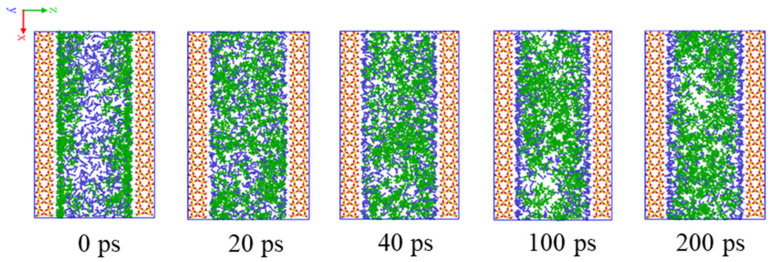
Snapshots of CO_2_ displacing hexane from the SiO_2_-OH wall process from the initial moment to 200 ps in the simulation box.

Figure 1 shows the time evolution of CO_2_ displacing hexane from the SiO_2_-OH wall at 10.5 MPa from the initial moment, *t* = 0 ps, to *t* = 200 ps. It is seen from Figure 1 that hexane molecules adsorb stably at the wall and form a uniform oil layer at the initial moment. This distribution is gradually disturbed as CO_2_ molecules diffuse into the area near the wall, occupy on the wall surface and displace hexane from the wall. As simulation time increases from *t* = 20 to *t* = 40 ps, the displacing process is clearly exhibited. More and more CO_2_ molecules displace hexanes and adsorb on the wall surface. When it evolves to 100 ps, the displacing process approaches completion, and remains little difference when evolving to 200 ps. The supercritical CO_2_ molecules almost completely occupy the wall surface and form several thin gas layers. As the result of CO_2_′s strong dissolving capacity, the free hexanes are mixed well with CO_2_ and no clusters are formed in the shale slit.

**Figure 2:**
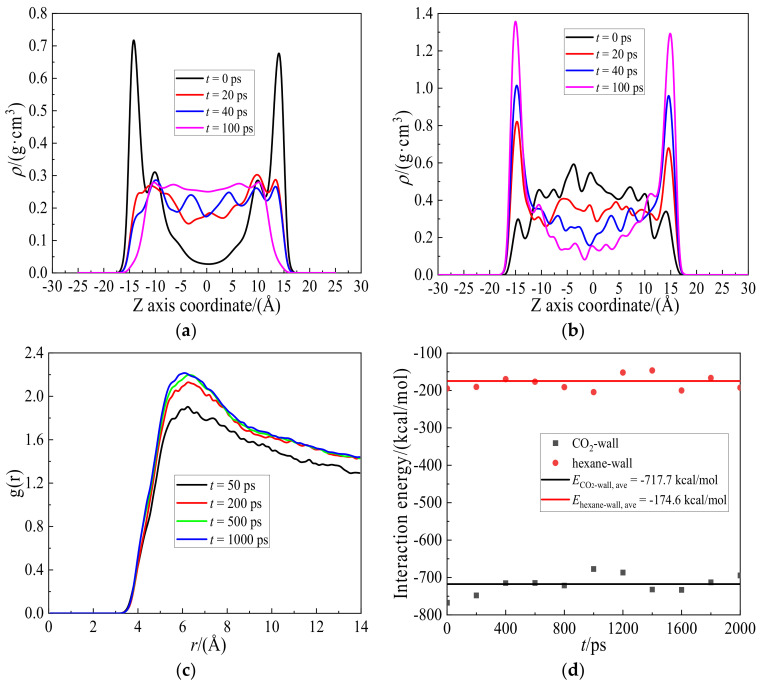
Density distributions of (**a**) hexane and (**b**) CO_2_ along the Z axis direction from the initial moment to 100 ps. (**c**) Radial distribution functions, g(*r*), of carbon-to-carbon atoms in CO_2_, C-C, from 50 to 1000 ps. (**d**) Variations of interaction energies of hexane-wall and CO_2_-wall with time from initial moment to 2000 ps.

To analyze the displacing process in detail, the density distributions of hexane and CO_2_ along the Z direction with time were calculated and are shown in Figure 2a,b. At the initial moment (*t* = 0 ps), the density of hexane exhibits two symmetrical peaks about 0.73 g/cm^3^ near the wall. The two-layer hexanes adsorb firmly on the wall surface, and form two stable oil layers. However, the hexane density decreases to nearly 0.05 g/cm^3^ at the center location. This huge distribution difference gradually diminishes as simulation time reaches 100 ps. At this time, the hexane molecules distribute uniformly in the bulk region, while the density gradually decreases to near zero when approaching the wall. This can be understood that hexane molecules are displaced by CO_2_ thoroughly from the wall, while the density of CO_2_ approaches to the maximum. It can be seen from Figure 2b that the evolution of CO_2_ density distribution with time is opposite to that of hexane. The density variation trends of CO_2_ and hexane have explicitly elucidated the procedure of CO_2_ displacing hexane adsorbed on the shale wall, which is the same as the snapshots shown in Figure 2.

Figure 2c shows radial distribution functions, g(*r*), of carbon-to-carbon atoms, C-C, in CO_2_ molecules from 50 to 1000 ps. It can be seen from Figure 2c that the peak values of g(*r*) decrease with time, indicating that the distribution for CO_2_ at 6.2 Å becomes less tight as time evolves. As the displacing process continues, CO_2_ molecules distribute widely in the whole system, resulting in fewer CO_2_ molecules around each other. Due to strong interactions between the CO_2_ and SiO_2_-OH wall, some CO_2_ molecules adsorb and gradually aggregate on the wall surface, resulting in slightly denser distribution. In this study, the interaction energy between CO_2_ and the wall drives hexane away from the wall.

Figure 2d shows the variations of interaction energies for the hexane-wall and the CO_2_-wall with time from the initial moment to 2000 ps. The interaction energies of the hexane-wall and CO_2_-wall, *E*_hexane-wall_ and *E*${\mathrm{CO}}_{2}$_-wall_, are stabilized at −174.6 and −717.7 kcal/mol respectively and kept almost constant in the simulation process. The stronger interactions of the CO_2_-wall in comparison with the hexane-wall’s render CO_2_ preferentially occupy hexane’s locations on the wall surface and displace hexane from it. Plenty of hexanes are driven to fall into the shale slit and mix with CO_2_ molecules. Therefore, the whole displacing process is completed.

### 2.2. Effect of CO_2_ Injection Pressure on Hexane Displacement

**Figure 3:**
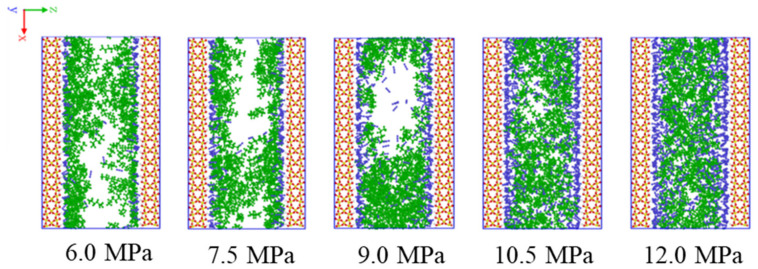
Variations of snapshots of CO_2_ displacing hexane from the SiO_2_-OH wall at the equilibrium state with injection pressures from 6.0 to 12.0 MPa.

Figure 3 shows variations of snapshots of CO_2_ displacing hexane from the SiO_2_-OH wall at the equilibrium state with injection pressures from 6.0 to 12.0 MPa. As shown in Figure 3, the amount of CO_2_ adsorbed on the wall increases with pressures from 6.0 to 9.0 MPa, and keeps almost constant at pressures higher than 9.0 MPa. The increasing and then nearly constant trends show unsaturated and saturated adsorption states for CO_2_ on the wall, resulting in the increasing and constant thickness of the adsorption layer respectively. The limited adsorption capacity of the SiO_2_-OH wall cannot hold more CO_2_ molecules after saturated adsorption, allowing some desorbed ones to diffuse in the shale slit, thereby destroying the cluster structure that may have formed previously.

**Figure 4:**
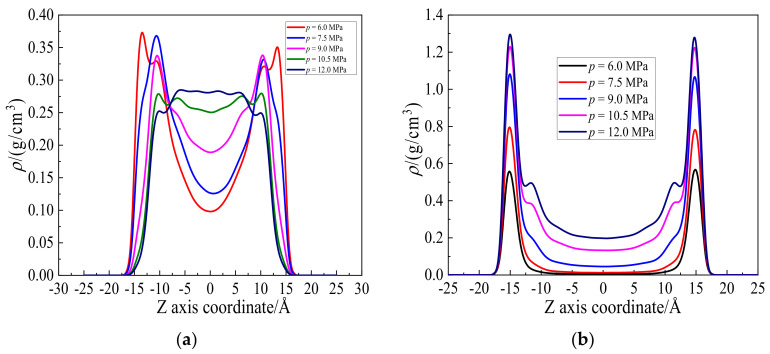
Density distributions of (**a**) hexane and (**b**) CO_2_ along Z axis direction at pressures from 6.0 to 12.0 MPa.

Figure 4 shows the density distributions of hexane and CO_2_ along the Z axis direction at pressures from 6.0 to 12.0 MPa. The density of hexane near the wall and in the shale slit gradually decreases and increases as pressure increases from 6.0 to 12.0 MPa, respectively. The uniform density distribution along Z direction of hexane is opposite to CO_2′_s at higher pressure in Figure 4b, as revealed in the previous section. At low pressure, there are still some hexanes remaining on the unsaturated wall surface. As pressure increases, more CO_2_ molecules adsorb on the wall and displace the rest of the hexanes from the wall. The hexanes are pushed into the shale slit, while the CO_2_ molecules are aggregated on the wall. Therefore, the densities of hexanes and CO_2_ molecules exhibit opposite trends, in agreement with the snapshots in Figure 3.

**Figure 5:**
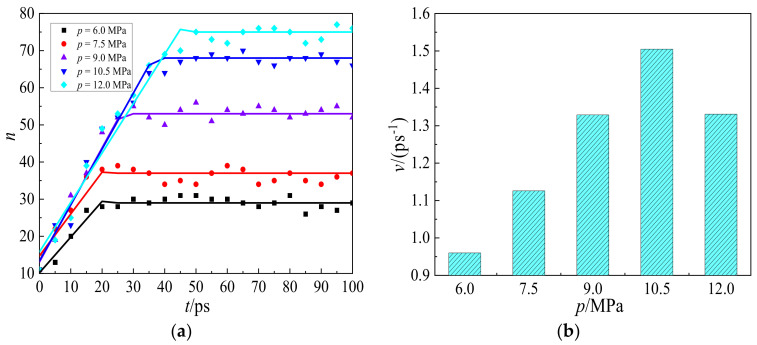
(**a**) Variations of the number of hexanes, *n*, displacing from the wall with time, *t*, from the initial moment to 100 ps, and (**b**) the displacing velocity of hexanes, *v*, at pressures from 6.0 to 12.0 MPa. The displacing velocity was obtained from the linear area in Figure 5a.

To quantificationally analyze the displacement performance, Figure 5 illustrates the time evolution of the number of hexanes being displaced from the wall by CO_2_ injections with different pressures. It can be seen from Figure 5a that the number of hexanes departing from the wall, *n*, increases linearly with time, *t*, and remains almost constant after a certain time frame. By linearly fitting the displacing number, *n*, with time *t*, *n*~*t*, in the linear area the displacing velocity, *v*, was obtained and shown in Figure 5b. The displacing velocity increases from 0.96 to 1.51 ps^−1^, when CO_2_ injection pressure increases from 6.0 to 10.5 MPa, and then decreases to 1.33 ps^−1^ at 12.0 MPa. As pressure increases from 6.0 to 10.5 MPa, CO_2_ molecules displace hexane from the wall more rapidly due to enhanced adsorption. However, the hindrance effect is enhanced as pressure increases to 12.0 MPa. Some unabsorbed CO_2_ molecules in the shale slit can be a hindrance to hexane diffusion, which could slow down the displacing velocity of hexane. Therefore, the optimal pressure in the displacing velocity is 10.5 MPa.

**Figure 6:**
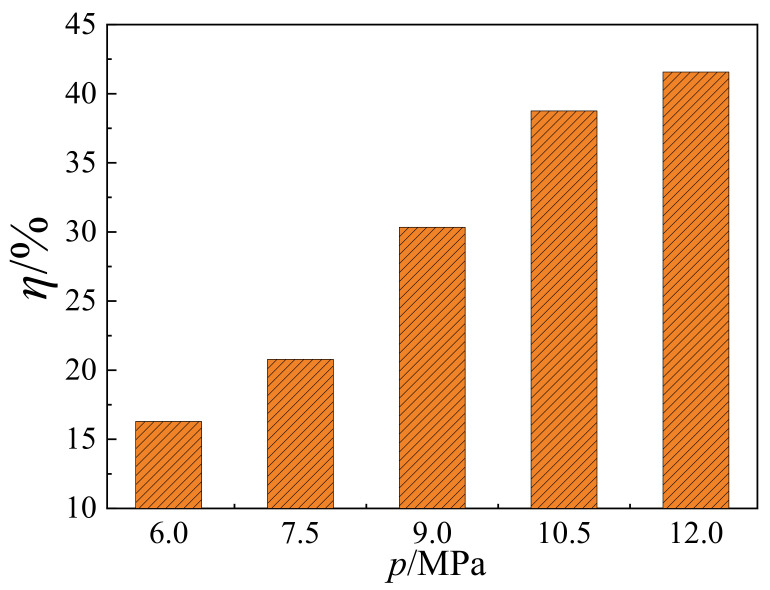
Variations of displacing efficiency for hexane, *η*, with pressures from 6.0 to 12.0 MPa.

The displacing velocity, *v*, cannot directly tell the recovering efficiency under the practical conditions, which is essential to unconventional shale oil production industry. To address this problem, the displacing efficiency, *η*, is accordingly proposed and defined as,

(1) $$\eta =\frac{n}{N}$$

where, *n* and *N* represent the number of hexanes displacing from the wall and the total number of hexanes in the system, respectively. According to Figure 5a, the number of hexanes displacing from the wall, *n*, keeps almost constant at equilibrium after 50 ps. In that case, the number, *n*, was counted and averaged. As shown in Figure 6, the displacing efficiency of hexane, *η*, increases from 16.3% to 42.1%, when the injection pressure increases from 6.0 to 12.0 MPa. At lower pressure, the adsorption of CO_2_ in the shale slit pore is still far away from saturation. As the pressure increases further, additional CO_2_ molecules adsorb on the wall surface and displace the hexane from the wall surface for the enhanced adsorption affinity of CO_2_. Nevertheless, such an increasing trend of displacing efficiency slows down at higher pressure due to the steric hindrance effect in the shale slit. As shown in Figure 3 and Figure 4b, the shale slit is full of CO_2_ and hexane molecules, rendering it more difficult to further conduct the displacing process. Taking both displacing velocity and efficiency into consideration, the optimal injection pressure is 10.5 MPa.

### 2.3. Mechanisms of CO_2_ Injection Pressure on the Hexane Displacing Process

To reveal the underlying mechanisms of the displacing process, NEMD simulations were further conducted to analyze the binary mixture diffusion. The force, Γ*_ex_*, exerting on the molecules to drive the fluid flow can be expressed as,

(2) $$\Gamma_{ex}=-\frac{d\mu }{dz}=-k_{B}T\frac{d\ln f}{dz}=k_{B}T\frac{\ln(f_{1}/f_{2})}{L_{x}}$$

where, *k_B_*, *T*, *f*_1_, *f*_2_, and *L_x_* are Boltzmann constant, temperature, inlet fugacity, outlet fugacity, and length of the simulation box, respectively. According to the relationship between the external force and net flux, the Onsager coefficient, *L_ij_*, can be obtained and expressed as,

(3) $$\left[\begin{array}{c} j_{1}\\ j_{2} \end{array}\right]=-\left[\begin{array}{cc} L_{11} & L_{12}\\ L_{21} & L_{22} \end{array}\right]\left[\begin{array}{c} \frac{\partial \mu_{1}}{\partial z}\\ \frac{\partial \mu_{2}}{\partial z} \end{array}\right]=\left[\begin{array}{cc} L_{11} & L_{12}\\ L_{21} & L_{22} \end{array}\right]\left[\begin{array}{c} \Gamma_{ex1}\\ \Gamma_{ex2} \end{array}\right]$$

here, *j_i_*, *L_ii_*, and *L_ij_* are net flux of component *i*, Onsager coefficients of diagonal and off diagonal respectively. The net flux, *j*, can also be computed by equation, *j_i_* = *ρ_i_*·*v_com, i_*. Here, *ρ_i_* and *v_com, i_* are density and streaming velocity of component *i*, respectively. Γ*_ex_* is the external force exerting on the component *i*, and expressed as, $\Gamma_{ex,i}=-\partial \mu_{i}/\partial z$. The hexane and CO_2_ are marked as components 1 and 2 respectively in this study. We note that by only applying external force to single component *i* (i.e., Γ*_ex_*_,*i*_ > 0, Γ*_ex_*_,*j*_ = 0), the net fluxes can be obtained according to equation, *j_i_* = *ρ_i_*·*v_com, i_*. Accordingly, the Onsager coefficients *L*_11_ and *L*_22_ for hexane and CO_2_, can be extracted by *L*_11_ = *j*_1_/Γ*_ex_*_1_ and *L*_22_ = *j*_2_/Γ*_ex_*_2_, respectively. Meanwhile, the diagonal Onsager coefficients *L*_12_ and *L*_21_ are calculated as, *L*_12_ = *j*_1_/Γ*_ex_*_2_ and *L*_21_ = *j*_2_/Γ*_ex_*_1_, respectively.

**Figure 7:**
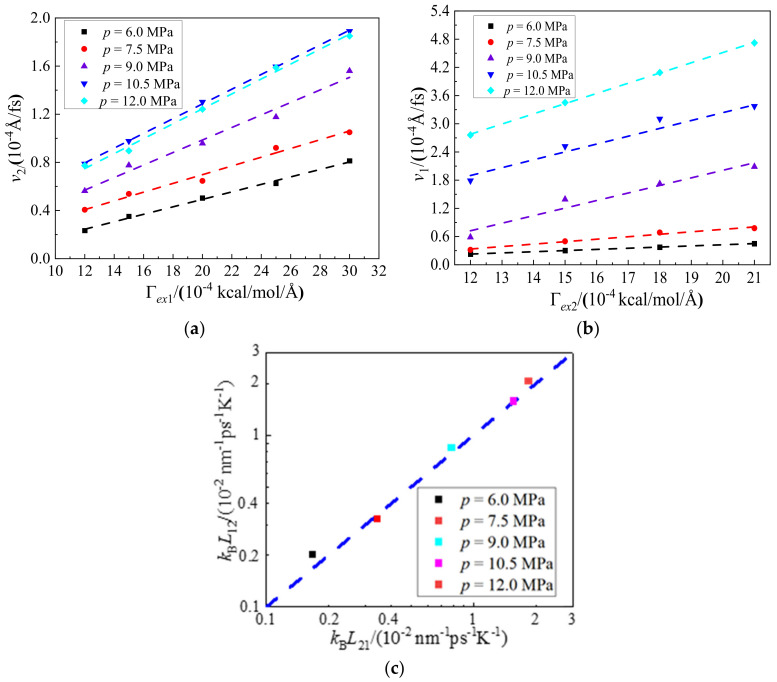
Variations of (**a**) CO_2_ velocity, *v*_2_, when external forces, Γ*_ex_*_1_, are applied on hexane, (**b**) hexane velocity, *v*_1_, when the other external forces, Γ*_ex_*_2_, are applied on CO_2_, and (**c**) diagonal Onsager coefficients *L*_12_ and *L*_21_, with injection pressures from 6.0 to 12.0 MPa. The blue dotted line, *y* = *x*, represents the isoline of the horizontal and vertical axes, *L*_12_ = *L*_21_.

As depicted in Figure 7a,b, the driven streaming velocities of hexane and CO_2_ exhibit good linear relationships with external forces. This further reveals that the diagonal Onsager coefficients are independent with external force. Figure 7c shows variations of diagonal Onsager coefficients with pressures from 6.0 to 12.0 MPa. It can be seen from Figure 7c that the diagonal Onsager coefficients, *L*_12_ and *L*_21_, exhibit good agreements with each other, and satisfy well with Onsager reciprocal relation. It can be inferred from this result that the mutual diffusion plays an essential role in the displacing process and will be revealed in detail in the following parts.

**Figure 8:**
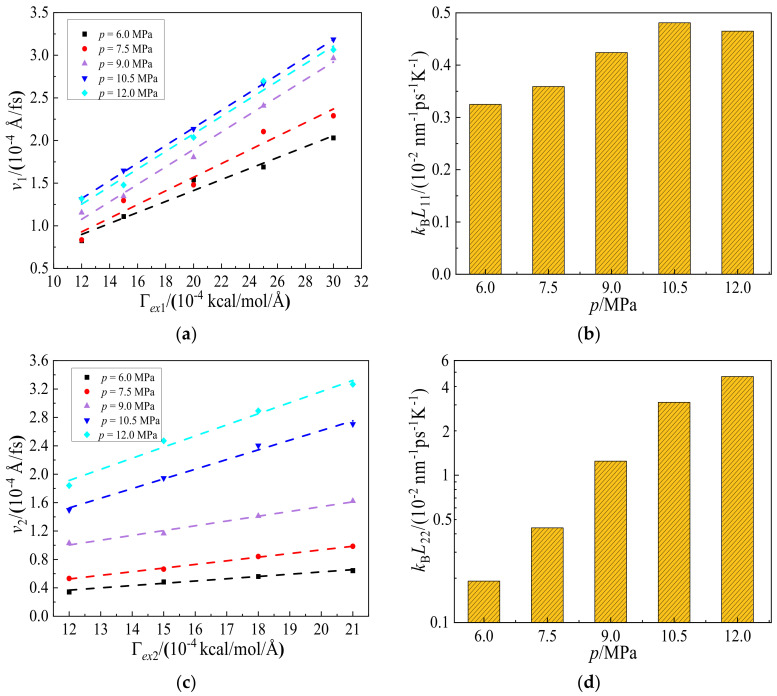
Variations of (**a**) velocity, *v*_1_, and (**b**) Onsager coefficient, *k*_B_*L*_11_, of hexane when external forces, Γ*_ex_*_1_, are applied on hexane, (**c**) velocity, *v*_2_, and (**d**) Onsager coefficient, *k*_B_*L*_22_, of CO_2_ when the other external forces, Γ*_ex_*_2_, are applied on CO_2_, at pressures from 6.0 to 12.0 MPa with 1.5 MPa internals, respectively.

The velocities, *v*, and Onsager coefficients, *L_ii_*, for hexane and CO_2_, at pressures from 6.0 to 12.0 MPa are shown in Figure 8. It can be seen from Figure 8a that the velocity of hexane increases linearly with force. We can further see that the Onsager coefficients of hexane increase from 0.325 × 10^−2^ to 0.481 × 10^−2^ nm^−1^ps^−1^K^−1^, when the pressure of CO_2_ increases from 6.0 to 10.5 MPa. Nevertheless, the Onsager coefficient of hexane decreases after that and slightly reduces to 0.465 × 10^−2^ nm^−1^ps^−1^K^−1^ at the pressure of CO_2_ being 12.0 MPa. It should be pointed out that the injected sCO_2_ not only displaces the oil from the shale wall, evident from Figure 8, but also generally enhances the diffusion rate of oil (hexane). That is to say, the presence of sCO_2_ will render it easier for oil to leave out the shale channel. In combining these two effects, one can expect that the sCO_2_ displacing oil method will achieve high recovery efficiency while reducing the oil exploiting time.

Meanwhile, it can be seen from Figure 8d that the Onsager coefficient of CO_2_ increases rapidly with pressure. At lower pressures (6.0 and 7.0 MPa), CO_2_ molecules diffuse slowly due to a larger number of them adsorbing on the wall surface. With increasing pressure to 9.0 and 10.5 MPa, the diffusion of CO_2_ molecules accelerates sharply. At 12.0 MPa, the Onsager coefficient of CO_2_ is 4.68 nm^−1^ps^−1^K^−1^, which is over an order of magnitude higher than that of hexane, 0.465 nm^−1^ps^−1^K^−1^. However, the highest pressure corresponding to the highest diffusion for CO_2_ molecules reduces hexane diffusion, which can reduce shale oil recovery efficiency. In consideration of the displacing velocity, the optimal injection pressure is 10.5 MPa.

**Figure 9:**
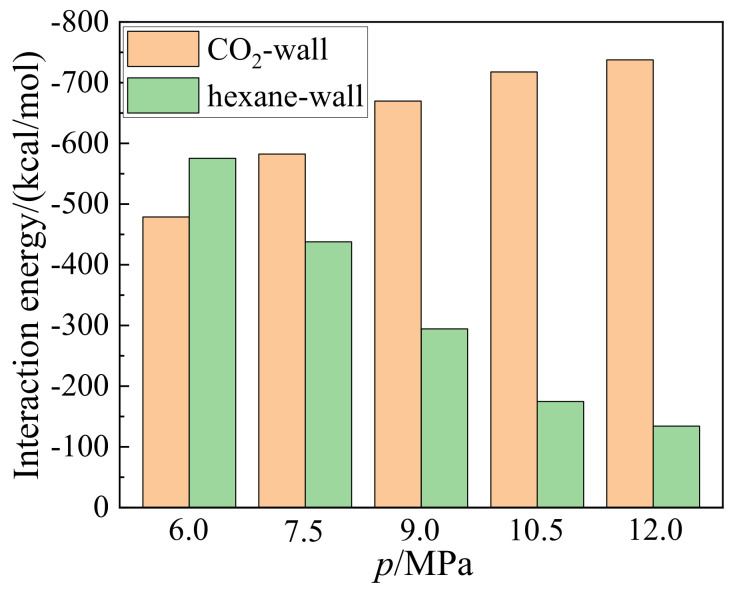
Variations of interaction energies of hexane-wall and CO_2_-wall with pressures from 6.0 to 12.0 MPa with 1.5 MPa internals.

Figure 9 shows the interaction energies of the hexane-wall and CO_2_-wall with pressures from 6.0 to 12.0 MPa. It can be seen from Figure 9 that the interaction energies for the hexane-wall and the CO_2_-wall decrease and increase with pressure in the range studied. For instance, the interaction energies for the CO_2_-wall increase from −478.8 to −737.6 kcal/mol, while that for the hexane-wall decrease from −575.3 to −134.2 kcal/mol, when the pressure increases from 6.0 to 12.0 MPa. At higher pressure, CO_2_ molecules distribute more densely near the wall, leading to stronger CO_2_-wall interaction energies, which is similar to the results given in previous studies [26,27]. The dominant interaction energies shift from hexane-wall to CO_2_-wall at 7.5 MPa, which shows that CO_2_ molecules are more inclined to adsorb on the wall surface and displace hexane from the surface. It can be understood that the further distance between the hexane and the wall weakens the interactions between them according to Equation (2). On the other hand, the stronger CO_2_-wall interactions drive more hexane molecules away from the wall, resulting in most hexane distributing in the bulk region, which is in agreement with the results in the previous part.

## 3. Materials and Methods

### 3.1. Model and Configurations

**Figure 10:**
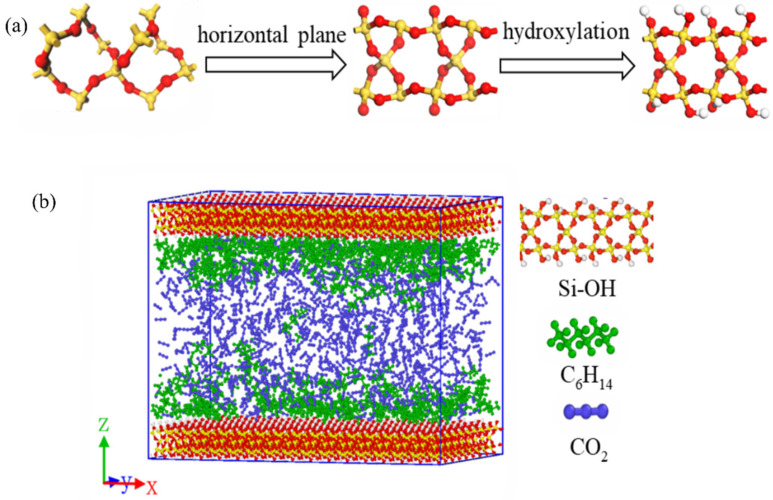
(**a**) Hydroxylation process of silica, SiO_2_-OH, and (**b**) Simulation setup. The walls at the upper and lower edges are hydroxylated silica, SiO_2_-OH, in which the red, yellow, and white spheres represent oxygen, silica, and hydrogen atoms, respectively. The green and bule molecules in the shale slit are hexane (C_6_H_14_) and carbon dioxide (CO_2_) respectively.

The simulation setup is comprised of three sections, including two hydroxylated silica crystal (SiO_2_-OH) walls located at the upper and lower edges of the box, creating a channel with the flowing direction being the *x* axis, as shown in Figure 10b. The chemical structure of the shale wall, SiO_2_-OH, is illustrated in in Figure 10a. The dimensions of the simulation box, *L_x_* × *L_y_* × *L_z_*_,_ are set as 78.56 × 48.62 × 50 Å^3^. There are full of chain alkane such as hexane, n-decane, and tetradecane in nano scale shale. Among these alkanes, hexane accounts for a large percentage and has many typical properties. In addition, hexane can well reflect the performances of shale oil in the nanochannel and can work as a representative in extensive studies [18,23,28]. Therefore, hexane was the adopted and represented oil in this study. A snapshot of the integrated simulation system is depicted in Figure 10b.

The simulations were performed as follows. Firstly, the process of CO_2_ displacing hexane was conducted. In that case, the movement and density distribution of CO_2_ and hexane were discussed. Secondly, the effects of injection pressures of CO_2_, which range from 6.0 to 12.0 MPa with 1.5 MPa intervals, on the displacing process were investigated to figure out the optimal pressure, evaluated by displacing velocity and efficiency. Finally, the corresponding underlying mechanisms of the displacing process were further explored.

### 3.2. Force Fields of Adsorbent and Adsorbates

Adsorbate-adsorbate, adsorbent-adsorbate interactions were both explicitly considered in our simulations. Initially, hexane molecules were described by OPLS-AA force fields [29], which are comprised of bond contraction energy, *E_bonds_*, angular bending energy, *E_angle_*, dihedral angle torsion energy, *E_torsions_*, van der Waals interaction energy, *E_vdW_*, and electrostatic interaction energy, *E_ele_*, expressed as,

(4) $$E_{all}=E_{bonds}+E_{angles}+E_{torsions}+E_{vdW}+E_{ele}$$

The detailed parameters of the OPLS-AA model for hexane are shown in Table 1, Table 2, Table 3 and Table 4. The flexible CO_2_ molecule was represented by the EPM2 model [30], with detailed parameters being listed in Table 1, Table 2 and Table 4. In addition, CLAYFF force fields were adopted to describe the SiO_2_-OH wall [31] given in Table 1, Table 2 and Table 4. The force fields of species, including hexane, CO_2_ and SiO_2_-OH, adopted in this work have been verified to be effective in adsorption and diffusion in extensive studies [18,19,32].

In addition to the bonded interactions, the nonbonded ones, including short-range (Lennard-Jones 12–6) and long-range force fields are also summed to describe interactions among atoms, expressed as,

(5) $$U=4\varepsilon \left[{\left(\frac{\sigma }{r_{ij}}\right)}^{12}-{\left(\frac{\sigma }{r_{ij}}\right)}^{6}\right]+\frac{q_{i}q_{j}}{4\pi \varepsilon_{0}r_{ij}}$$

herein, *ε* and *σ* are potential well depth and collision distance, respectively. The LJ parameters between two unlike species were calculated from the Lorentz-Berthelot mixing laws [33], following

(6) $$\varepsilon_{ij}=\sqrt{\varepsilon_{i}\varepsilon_{j}}$$

(7) $$\sigma_{ij}=\frac{\sigma_{i}+\sigma_{j}}{2}$$

### 3.3. Simulation Configurations

The equilibrium and nonequilibrium molecular dynamics (EMD and NEMD) simulations were conducted using LAMMPS packages [34], starting with the initial configurations obtained from grand canonical Monte Carlo (GCMC) simulations that were conducted at 353.15 K throughout the simulations and targeted pressure using DL_MONTE simulation packages [35]. The NEMD simulations were performed by exerting external force on one of the two components in the presence of the sCO_2_ and hexane mixtures. The Nosé-Hoover thermostat was applied to maintain the temperature of the system with a damp coefficient of 100 fs. In all simulations, the cutoff radius equaling 14.0 Å was applied to truncate short-range and long-range interactions in the real space. The Ewald method with a tolerance of 1.0 × 10^−4^ was adopted for the long-range corrections to the coulomb interactions. The time step was set as 1.0 fs and periodic boundary conditions were applied in all three directions. The simulations were performed for 4.0 ns in the canonical ensemble with the first 2.0 ns for equilibration and the remaining 2.0 ns for statistical analysis at a frequency of 500 steps. The temperature of the system remains at 353.15 K as it is the critical temperature of sCO_2_. We computed the density and number of CO_2_ molecules in the simulation system using grand canonical Monte Carlo simulations (GCMC) at different pressures, which are listed in Table 5. In our simulations, the number of hexane molecules is 178. Indeed, the number of hexanes remains constant in this work. This is done for the two reasons: (1) In practice, the sCO_2_ molecules are injected into the shale oil reservoir, with the temperature and pressure of the shale oil reservoir being fixed, which leads to a fixed content of oil molecules for a given reservoir. Therefore, it is justified to investigate the efficiency of sCO_2_ dispatching the oil by fixed the molecule numbers adsorbed on the shale wall. (2) The second reason is for controlling the variable without neglecting the essential performance. Although the hexane density can be varied at different pressures (i.e., different depths of the reservoirs), the displacing process is basically unchanged. To elucidate the process and its underlying mechanisms more clearly, some necessary simplifications were conducted in this work. This treatment can not only reveal the main mechanisms and characteristics, but also save computation costs. Therefore, the number of hexane molecules remains constant in this work.

### 3.4. Verification of the Simulation

**Figure 11:**
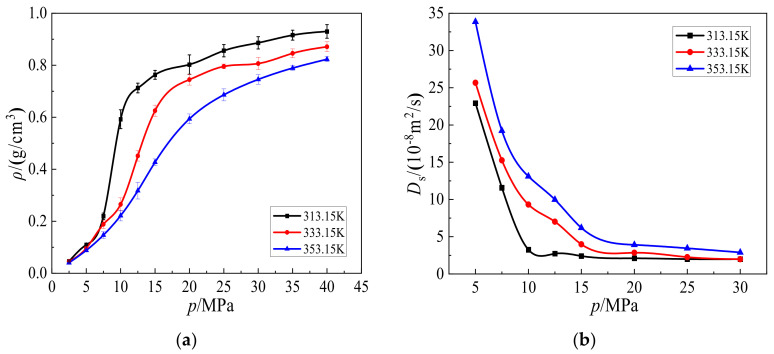
Variations of (**a**) density and (**b**) self-diffusion coefficient for CO_2_ at different temperatures and pressures from 6.0 to 12.0 MPa.

To confirm the accuracy and reliability of this work, the potentials were verified. The density and self-diffusion coefficient for CO_2_ represented by the EMP2 model at different temperatures and pressures were calculated and shown in Figure 11a,b, respectively. It can be seen from Figure 11a,b that the CO_2_ density and self-coefficient are close to the results in the database of the American National Standards Institute. Indeed, the standard error between the two is within 6.0%, matching well with each other. As shown in Figure 11b, the self-diffusion coefficients of CO_2_ decrease/increase with pressure/temperature in a wide range, which caters well to the trend reported in previous studies [36,37].

## 4. Conclusions

Molecular dynamics simulations were performed to explore process of CO_2_ displacing hexane from SiO_2_-OH wall, the effects of CO_2_ pressure on the displacing velocity and efficiency as well as the underlying oil recovering mechanisms in binary mixtures are discussed in detail. The displacing process can be divided into three steps; first, supercritical CO_2_ molecules fill in the shale slit, and then adsorb on the wall surface and mix with hexane, resulting in a loose hexane layer, and they finally displace the hexane from the wall due to stronger interactions between CO_2_ and the wall. The displacing velocity increases to a maximum at 10.5 MPa, and then decreases with pressure. In addition, the displacing efficiency increases from 16.3% to 42.1% when pressure increases from 6.0 to 12.0 MPa. This phenomenon is caused by the change in diffusion circumstance, in which the Onsager coefficients of CO_2_ and hexane are correlated to increase with pressure, until the diffusion rate of hexane being suppressed by the highly dense distribution of CO_2_ molecules is equal to 12 MPa. Indeed, the rapid transportation of CO_2_ molecules in the binary components (CO_2_ and hexane) promotes the hexane diffusion, which is in favor of driving oil out of nanochannels. The interaction energies of the CO_2_ wall are stronger than that of the hexane wall when pressures are higher than 7.5 MPa, which originally accounts for the efficient hexane displacement. Taking the displacing velocity and efficiency and the hexane diffusion rate into consideration, the optimal injection pressure of CO_2_ is 10.5 MPa in this work. Our study provides detailed mechanisms of CO_2_ displacing hexane from a wall, which contributes to a deeper understanding of unconventional oil and gas exploitation and utilization.

## Figures and Tables

**Table 1:** Bond parameters of C_6_H_14_, CO_2_ and SiO_2_-OH adopted here.

Species	Bond Type	*K_r_* (kcal/mol)	*r*_0_ (Å)
C_6_H_14_	C-C	268.0	1.529
	C-H	340.0	1.09
CO_2_	C=O	615.322	1.149
SiO_2_-OH	O-H	554.1349	1.0

**Table 2:** Angle parameters of C_6_H_14_, CO_2_ and SiO_2_-OH.

Species	Angle Type	*K*_θ_ (kcal/(mol·rad^2^))	*θ* (°)
C_6_H_14_	C-C-C	58.35	112.70
	C-C-H	37.5	110.70
	H-C-H	33.0	107.80
CO_2_	O=C=O	295.411	180
SiO_2_-OH	Si-O-H	30.0	109.47

**Table 3:** Dihedral angle parameters of OPLS-AA model for C_6_H_14_.

Types	V_1_ (kcal/mol)	V_2_ (kcal/mol)	V_3_ (kcal/mol)	V_4_ (kcal/mol)
C-C-C-C	1.30	−0.05	0.30	0
C-C-C-H	0	0	0.30	0
H-C-C-H	0	0	0.30	0

**Table 4:** Lennard-Jones 12–6 potentials and charges of atoms.

Species	Atoms	*ε* (kcal/mol)	*σ* (Å)	*q* (e)
SiO_2_-OH	Si	1.84 × 10^−6^	3.302	+2.1
	O	0.1554	3.1655	−1.05
	O_OH	0.1554	3.1655	−0.95
	H_OH	0	0	0.425
C_6_H_14_	C_CH_3_	0.066	3.50	−0.18
	C_CH_2_	0.066	3.50	−0.12
	H_C_6_H_14_	0.030	2.50	0.06
CO_2_	C_CO_2_	0.0559	2.757	0.6512
	O_CO_2_	0.1559	3.033	−0.3256

**Table 5:** Variations of density and number of CO_2_ molecules at 353.15 K with pressures.

*p* (MPa)	*ρ* (g·cm^3^)	Number
6.0	0.14688	246
7.5	0.22847	394
9.0	0.3745	637
10.5	0.5551	961
12.0	0.6728	1173

## Data Availability

Not applicable.

## Sample Availability

Samples of the compounds are not available from the authors.
